# Impact of a Single-Day Lockdown on COVID-19: An Interrupted Time Series Analysis

**DOI:** 10.7759/cureus.17299

**Published:** 2021-08-19

**Authors:** Saif Aldeen AlRyalat, Khaled A Elubous, Ali D Al-Ebous, Azmi Mahafzah

**Affiliations:** 1 Ophthalmology, The University of Jordan, Amman, JOR; 2 Surgery, King Hussein Cancer Center, Amman, JOR; 3 Microbiology, The University of Jordan, Amman, JOR

**Keywords:** covid-19, lockdown, corona virus, jordan, time series analysis

## Abstract

This study evaluated a special form of lockdown that was applied in Jordan: one day of lockdown every week, which was applied on consecutive weekend days (i.e., Friday in Jordan, for 24 hours). We tried to assess the impact of this form of lockdown on the daily number of positive coronavirus disease 2019 (COVID-19) cases, using interrupted time series analysis. We included the period of March 5 to April 17, 2021, as the period affected by the Friday lockdown, which was applied to seven consecutive Fridays with a total of 168 hours. We used R version 4.0.5 (R Foundation for Statistical Computing, Vienna, Austria) for our analysis. We used Poisson model regression analysis, where the number of positive cases was used as the outcome variable, while the total number of tests, time, and lockdown were used as the predictor variables. We further performed quasi-Poisson regression analysis to confirm the first model. On Poisson model regression analysis, it was found that there was an evidence of an increase in the number of positive COVID-19 cases following the intervention of Friday lockdown, with a p value of <0.001 (relative risk, 1.569; 95% confidence interval, 1.549-1.590). On using quasi-Poisson regression, similar results were found with a wider confidence interval. We concluded that a single weekend day lockdown led to an increase in the number of daily cases of COVID-19. Therefore, we recommend authorities to adhere to evidence-based measures or to the WHO recommendations in the dealing with this pandemic.

## Introduction

Coronavirus disease 2019 (COVID-19) was first reported in Wuhan, China, on December 12, 2019, and since then, countries have imposed several restrictive measures to limit the spread of the infection [1]. Initially applied in China, a complete lockdown has been shown to be an important measure to control the spread of the disease [2]. Other countries modified the complete lockdown to restrict movement during certain times or applied it on certain activities. One such modification is a curfew after 6 pm, which was adopted in France, and a recent study found that this led to the opposite effect, that is, an increase in the number of COVID-19 cases instead of controlling the disease [3]. The reason behind this opposite effect of such partial lockdowns might have been the overcrowding of people in a shorter period of time in more confined places [4]. Another form of partial lockdown that had never been studied was imposed in Jordan, which was a single-day lockdown on Fridays.

After a few cases of confirmed severe acute respiratory syndrome coronavirus 2 (SARS-CoV-2) infection, Jordan authorities imposed very strict lockdown measures on March 17, 2020 [5]. However, after several months of the outbreak, they decided to mitigate the measures and adopted a different strategy of social distancing that avoided a strict or prolonged lockdown. This strategy included (1) imposition of a daily night lockdown (six hours) from 12 am to 6 am, (2) using distance learning for studying in the schools and universities, (3) increasing community awareness of hygiene as well as forcing people to wear masks in public places, and (4) preventing mass gatherings. In addition to that, an imposition of one weekend day of lockdown was placed. On that lockdown day, every city ​​activity, shops, and public places were to be closed. Furthermore, going out of the house was prohibited, except for those who held a permit such as healthcare personnel. This lockdown was applied to the whole country without an exemption. However, during other days of the week, there was no lockdown except for the six-hour period after the midnight (from 12 am to 6 am), and there was no restriction on people's movement during the rest of the day [6].

The non-pharmaceutical measures to deal with the COVID-19 outbreak include but are not limited to lockdown, home quarantine of infected people and their contacts, social distancing, face mask wearing and increasing community awareness about good hygiene [1,7]. One of the World Health Organization (WHO) key guiding principles on adjusting public health and social measures (PHSM) in the context of COVID-19 is to adopt measures with proven effectiveness, which should be evaluated through an evidence-based assessment and active monitoring of the impact of these implemented measures [8]. In order to evaluate the efficacy of social distancing measures, including lockdown, many modeling studies have been carried out [9]. Moreover, a plenty of interrupted time series (ITS) analyses have evaluated the efficacy of lockdown in haltering the pandemic breakout [10,11]. However, to our knowledge, there is no previous analysis that evaluated the efficacy of a weekly lockdown. Thus, the aim of this study was to evaluate the efficacy of this form of lockdown in the daily number of COVID-19 cases in Jordan, through an ITS analysis.

## Technical report

Methods and materials

**Figure 1:**
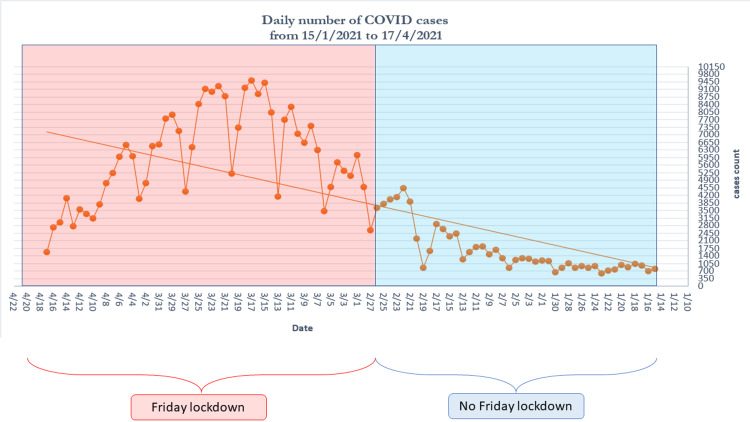
Daily number of COVID-19 cases in the included period in the analysis, from January 15 to April 17, 2021 Each orange-colored dot represents the corresponding count of daily cases. The period highlighted in blue color represents the pre-intervention (no Friday lockdown) and that highlighted in red represents the intervention period (Friday lockdown).

Data were derived from the Johns Hopkins University website [5]. The daily number of cases from January 15 to April 17, 2021, is shown in Figure 1. It includes the second wave of the COVID-19 pandemic in Jordan; the peak number of cases was on March 17, with a total of 9535 cases [5]. Effective on Friday, February 26, 2021, Jordanian authorities imposed a single day lockdown every week on Friday, the first weekend day in Jordan. Our analysis is based on the assumption that the number of new cases will change in a specific trend without an intervention. This pattern will change if an intervention is applied, and the magnitude of this change can be assessed using ITS regression analysis. For the analysis to be accurate, no other intervention should have been implemented during the included period; therefore, we selected two sequential periods, the first one (pre-intervention) from January 15, 2021, to February 25, 2021, where only mitigated measures where applied that included (1) imposition of a daily night lockdown (six hours) from 12 am to 6 am, (2) using distance learning for studying in the schools and universities, (3) increasing community awareness of hygiene as well as forcing people to wear masks in public places, and (4) preventing mass gatherings like parties or weddings.

The following period (intervention) was from February 26 to April 17, 2021; during this period, the same formerly mentioned measures were continued to be applied; however, the Friday lockdown was added. The chosen period has the advantage of no other interventions being changed except for the Friday lockdown. Figure 1 represents the two periods: the pre-intervention period (no Friday lockdown) highlighted in blue color and the intervention period (Friday lockdown) highlighted in red color. The Friday lockdown was expected to have an impact on the number of new cases after seven days of the first Friday lockdown (i.e., delayed or lagged effect), which is the duration from exposure to symptom appearance and infection confirmation [12]. So we included the period from January 22 to March 4, 2021, as the period affected by the no-Friday-lockdown period. We included the period from March 5 to April 17, 2021, as the period affected by the Friday lockdown period. We used R version 4.0.5 (R Foundation for Statistical Computing, Vienna, Austria) for our analysis. We used Poisson model regression analysis, where the number of positive cases was used as the outcome variable, while the total number of tests, time, and lockdown were used as the predictor variables. As the observations are not independent of each other, which is an important assumption for Poisson regression, we further performed quasi-Poisson regression analysis to confirm the first model. The data used in this study were deposited in an open repository [13].

Results

A total of 86 days were included in the analysis, which were within the second wave of the coronavirus in Jordan, including a total of 42 days that were not affected by the Friday lockdown intervention, and 44 days affected by the Friday lockdown.

The mean number of cases during the lockdown period was 7311, ranging from 6472 to 8819. During the period when no Friday lockdown was applied, the mean number of daily cases was 2266, ranging from 608 to 6068. Table 1 details the number of cases between each period.

**Table 1:** Characteristics of each lockdown period in terms of number of cases

Daily case parameters	Period of Friday lockdown	Period of no Friday lockdown
Mean	6139	2266
Median	6463	1608
Range	1596-9535	608-6068
Quartiles	4129-7968	1076-3455

On using Poisson model regression analysis, it was found that there was an evidence of an increase in the number of positive COVID-19 cases following the intervention of Friday lockdown, with a p value of <0.001 (relative risk, 1.569; 95% confidence interval, 1.549-1.590). Similar results were found by the quasi-Poisson regression, however, with a wider confidence interval. In conclusion, a single weekend-day-lockdown might increase the number of daily cases of COVID-19.

## Discussion

Many strategies of lockdown have been proposed, ranging from a strict full lockdown to mitigation-only measures. Chowdhury et al. proposed three patterns of lockdown for low- and middle-income countries, namely zonal, on-off and sustained mitigation, each of which could be imposed according to many factors including country healthcare infrastructure, economic considerations, and ability to maintain the strategy [14]. Several strategies of lockdown have been adopted by different countries. While some countries adopted a strict lockdown as in China and Lebanon, others adopted a more flexible or partial lockdown as in Sweden and the Netherlands [1,2].

Although randomized clinical trials (RCTs) are the gold standard in the evaluation of an intervention, many factors may limit our ability to conduct such a type of study. These factors include the high cost of an RCT and the difficulty in randomization or control of groups. These limitations are of particular concern during the evaluation of health policies or interventions that involve the whole community, for example, the health measures implemented by governments during epidemics. ITS analyses are of particular advantage in such circumstances [15].

A limited number of ITS analyses have been conducted in this regard, and all of them showed that social distancing is effective in reducing the number of cases related to COVID-19 [16,17]. Silva et al. found a statistically significant reduction in new confirmed cases in different state capitals in Brazil after a period of lockdown ranging from 13 to 23 days [16]. Tobías et al. also reported a reduction in daily cases and ICU admission after the implementation of strict measures in Italy and Spain [17].

However, in this analysis we did not find a statically significant effect of one-day-a-week lockdown on the reduction of daily cases. We suggest several explanations for this observation. Lockdown for one day in a week may change the social behaviors. For example, the process of citizens leaving their homes to fulfill their various needs will be distributed over six days instead of seven days, and thus, this may lead to overcrowding in the public places and shops during the off-lockdown days [4]. Another theoretical explanation is that unlike the long-term lockdown, the once-a-week lockdown does not help in achieving the goal of social distancing if other days of the week are available and social visits are possible in the near term of time. What may happen is shifting of the meeting dates from the lockdown day.

While our analysis provided an evidence of an increase in the number of daily COVID-19 cases with the weekly single-day lockdown, still further studies are needed to confirm our results before applying these findings. Several limitations need to be considered upon interpreting our results, including the small number of daily tests performed in Jordan, which might not be adequate to represent the actual number of daily cases. However, the stability of the daily testing pattern is the main pre-requisite for our time series analysis to be performed. Moreover, with the emergence of new variants that have different incubation periods, our pre-specified window between infection and diagnosis might also be different accordingly, despite the minimal impact it would impose on our results.

## Conclusions

This study evaluated a special form of lockdown that had been applied in Jordan that is one day of lockdown applied on consecutive weekend days (i.e., Fridays). We concluded that a single weekend-day lockdown might increase the number of daily cases of COVID-19. We recommend the decision makers and authorities to adhere to evidence-based measures or to the WHO recommendations in dealing with this pandemic. We also recommend further research to evaluate the implemented measures to give strong evidence through continuous feedback monitoring.
